# Genomes of Two Clinical Isolates of *Mycobacterium tuberculosis* from Odisha, India

**DOI:** 10.1128/genomeA.00199-14

**Published:** 2014-03-20

**Authors:** Mohammad Majid, Narender Kumar, Asifa Qureshi, Priyadarshini Yerra, Ashutosh Kumar, Mandala Kiran Kumar, Suma Tiruvayipati, Ramani Baddam, Sabiha Shaik, Aparna Srikantam, Niyaz Ahmed

**Affiliations:** a Pathogen Biology Laboratory, Department of Biotechnology and Bioinformatics, University of Hyderabad, Hyderabad, India; b Blue Peter Research Centre (Lepra Society), Cherlapally, Hyderabad, India; c Institute of Biological Sciences, Faculty of Science, University of Malaya, Kuala Lumpur, Malaysia; d Environmental Genomics Division, CSIR–National Environmental Engineering Research Institute (NEERI), Nagpur, India

## Abstract

We report whole-genome sequences of two clinical isolates of *Mycobacterium tuberculosis* isolated from patients in Odisha, India. The sequence analysis revealed that these isolates are of an ancestral type and might represent some of the “pristine” isolates in India that have not admixed with other lineages.

## GENOME ANNOUNCEMENT

Tuberculosis caused by *Mycobacterium tuberculosis* is a chronic infectious disease that is often fatal if not effectively treated. Every year, 8 to 9 million new infections and a death toll of 1.5 million are recorded worldwide. It is estimated that about one-third of the human population is infected with *M. tuberculosis* (1). Comparative genomic studies have provided deeper insights into the genetic diversity and clonal architecture of *M. tuberculosis* (2). Recent studies conducted on isolates from India have shown that highly concentrated reservoirs of the ancestral *M. tuberculosis* lineages prevail in South and Central India (3–6). Only a limited number of *M. tuberculosis* genomes from India are sequenced. The whole-genome analysis of ancestral and modern lineages would facilitate deciphering of the genetic variability and evolutionary mechanisms of this obligate parasite. We describe the whole-genome sequences of two *M. tuberculosis* strains, NA-A0008 and NA-A0009, isolated in 2008 from patients in rural Odisha, India.

Genomic DNAs of both strains were isolated using the Qiagen kit method. Whole-genome sequencing was carried out on an Ion Torrent sequencing platform (Life Technologies). The process generated 3 million and 2.9 million reads amounting to 89× and 93× genome coverage for NA-A0008 and NA-A0009, respectively, with a mean read length of 250 bp. The reads after filtration were assembled into 280 and 310 contigs for NA-A0008 and NA-A0009, respectively, using the MIRA v.2 *de novo* assembler. These contigs were ordered and reoriented according to the *M. tuberculosis* CCDC 5180 genome using in-house written scripts. The resulting draft genomes were annotated using the RAST annotation server (7), and CDSs were validated by comparing outputs from EasyGene (8) and Glimmer (9), as done previously (10–13). The number of rRNA operons were predicted in both strains using RNAmmer (14), while tRNAscan-SE (15) was used to identify tRNA sequences. Artemis (16) was used to glean the genome statistics of both the strains. The genome sizes of NA-A0008 and NA-A0009 were 4,259,206 and 4,271,739 bp, with coding percentages of 89.4% and 89.3%, respectively. The G+C contents of both strains were high, as usually observed for the *M. tuberculosis* complex, 65.31% (NA-A0008) and 65.28% (NA-A0009). The two genomes, NA-A0008 and NA-A0009, were predicted to encode 4,400 and 4,453 CDSs with average lengths of 866 and 857 bp, respectively. Both of them contained a single rRNA operon and 45 tRNA genes.

The availability of these genome sequences would definitely complement the gene pool analysis of the Indian strains from different parts of the country. Besides this, comparative genomic analysis and phylogenetic study of these isolates with other *M. tuberculosis* strains might give us important insights into the biology and molecular epidemiology of this organism.

### Nucleotide sequence accession numbers.

The *M. tuberculosis* NA-A0008 and NA-A0009 whole-genome shotgun projects have been deposited in the GenBank database under the accession numbers ALYG00000000 and ALYH00000000, respectively. The BioProject designations for these projects are PRJNA168604 and PRJNA168605, respectively.
